# Opening Editorial - The Importance of the Humanities in Medical Education

**DOI:** 10.15694/mep.2018.0000140.1

**Published:** 2018-07-09

**Authors:** Jonathan McFarland, Irina Markovina, Trevor Gibbs

**Affiliations:** 1 Sechenov University; 2 AMEE

**Keywords:** Medical Humanities, MedEdPublish special issue

## Abstract

This article was migrated. The article was marked as recommended.

An Abstract was not necessary for this article.

## Introduction

“ *Physicians are poised at the interface between the scientific and lay cultures*”  ( Kleinman, 1988)

The world of healthcare is changing. As we look through the lens of a bio-psycho-social approach to healthcare we are frequently amazed at the speed of growth of the science behind the subject and the new innovations that occur in technological support and operative procedures, almost on a daily basis. Our social approach to healthcare is rapidly undergoing such change. We see the rise of patients (the e-patient ( Riggare, 2018)) coming into healthcare, already equipped with the information about possible diagnoses and their treatment and looking for confirmatory evidence of what they feel should be next steps in their management.  Whilst we are still in control, sometimes we feel that only happens when we can justify our actions from evidence-based research.

At the same time the number of people with complex, chronic or multiple conditions is increasing, many of who remain in and place increasing demands upon their social community. We are seeing an ageing population, and the total number of years people can expect to live in poorer health continues to rise.

Just as healthcare is changing, then the world of healthcare education must change in response. Changes are occurring in curricula; new teaching, learning and assessment methodologies are presented with rapidity in the increasing number of educational journals. New simulation technologies abound to bring student learning closer to reality whilst recognising patient safety.

Frenk and colleagues, however, in a seminal Lancet report, spoke of how “ *Professional education has not kept pace with these challenges [in healthcare delivery] largely because of fragmented, out-dated, and static curricula that produce ill-equipped graduates”  *( Frenk *et al*., 2010 *)*

But what about the social side of healthcare education?

In 2016, the Behavioural & Social Sciences Teaching in Medicine (BeSST) Sociology Steering Group produced a Core Curriculum for Sociology in UK Undergraduate Medical Education ( BeSST, 2018).

In an opening forward from that report Ronald Harden (Professor of Medical Education at the University of Dundee, UK) was quoted as saying

“ *We see now a renewed emphasis on an ‘authentic’ curriculum in medicine with a move from the ivory tower of the university to the real world of medical practice……. this report from BeSST illustrates how sociology can ensure that as teachers, practitioners and students we can have a better understanding of the human being”.*

One of the elements of the sociology core curriculum is Topic 3 -Experiences of Health, Illness, Disability and Healthcare, and suggests that the students’ learning outcomes are:

To be able to:

• Discuss factors influencing patients’ experiences of health care
• Demonstrate an understanding of the experience and the role of carers
• Explain the ways in which health and illness and disability shape identity
• Identify the social, physical and emotional impact of living with illness
• Apply an understanding of the patient experience to medical practice

Whilst the BesSST document spoke of the outcomes rather than the teaching methods, we believe as co-editors of this MedEdPublish theme that some of the opportunities for teaching these learning outcomes lie in the Humanities- looking backwards to look forward. Although not given to thinking that all the answers lie with the Humanities, we do believe this approach, that casts a reflective look at various art forms, can inspire in addressing the BeSST learning outcomes.

## Discussion

This special issue of *MedEdPublish *will concentrate on the Humanities in Medical Education. The Medical Humanities is not a new discipline, with the term first being used by George Sarton in the 1940’s in the pages of a journal ominously called ISIS ( Hurwitz and Dakin, 2009). However, we believe that its relevance now is stronger than ever. There are many definitions, but this seems as good and appropriate as any; “an interdisciplinary field concerned with understanding the human condition of health and illness in order to create knowledgeable and sensitive health care providers, patients, and family caregivers” ( Klugman, 2017). In most instances this insight into people (patients) can be gained through looking at art, seeing through the eyes of the subject or the artist, reading stories, poems and novels that describe the human form, physical and psychological, listening to music and reflecting on why the composer wrote that piece of music, in that way and at that time.

And why are the humanities so necessary for 21 ^st^ century medical education, what can they add, and how can students benefit? Critical but difficult questions with a diversity of responses; perhaps they can help students and all levels of practitioners develop their own valuesand their communication skills; to better understand the patient point of view and their approach to illness: to think and reflect on theirs and others experiences: to cope better under stress: to see things more critically and probably much more. But, of course, this maybe the tip of the iceberg, and we want to hear your particular suggestion(s).

Medical education seemingly can appear to concentrate on one perspective; how to train medical practitioners to look after their patients better, which. without doubt, is critical in todays’ healthcare arena. Medicine is now firmly set and triangulated between the patient, the practitioner and the carer; all are equally vulnerable. To quote Liao “the reality is that the practice of Medicine is a human one. Medicine is about people at their most intimate frontiers. It is also practised by people.” ( Liao, 2017).

Along these lines, a recent study has investigated how the humanities can help medical students combat stress, and burnout. The results were positive, and the study confirmed the association between exposure to the humanities and a higher level of positive qualities (e.g. wisdom, empathy, spatial skills) in the students. The authors state, “The humanities might actually provide an indispensable language for exploring that strange, nuanced, and often nonsensical land called the human condition”. ( Mangione *et al*., 2018) This is why the humanities need to play a part in medical education; we are dealing withhuman beings, and their *nonsensical* ideas and emotions. The human condition cannot be fully understood by scientists; indeed “Most clinicians are not scientists; they have a different responsibility – to attempt to relieve distress and suffering ….” ( Heath, 2016) We feel that by introducing the Humanities back into medical education we help to provide more tools for medical practitioners to deal with their patients and their problems.

The rapid advancement in medical technology has led to great leaps in terms of diagnosis and treatment, and doctors and healthcare practitioners are far better equipped than fifty or even twenty years ago. The question now is, can they give the patients what they really need? Are they being taught to do so? In a recent article, ( Ofri, 2018) says, “In moments of medical crisis, you need a doctor who can help you navigate uncertainty. When your body threatens mutiny and you are peering into the abyss, you want a doctor who has contemplated mortality in a deep way. You want a doctor who is unafraid to wrestle with ambiguity and nuance”.

## Conclusion

We understand that there is a great deal of debate surrounding the medical humanities, and that one of the main issues is how to introduce it into medical education. Should it be fully integrated into the medical curriculum; should it be an elective; is it a “soft skill”, as compared to the hard currency of scientific knowledge?

Through this MedEdPublish Themed Edition, we are looking for your ideas, your thoughts and your practical applications, about the Humanities and whether they have a place within modern medial education and if they have, how can they be used and developed. Although the topic is very broad, our outcomes can be easily as broad but with our aim being to open this debate across the disciplines, across the ages and across the countries.

There are many initiatives around the world working on introducing the humanities into the medical curriculum, more and more symposia and more associations developing. The world we live in is not unidimensional, and precisely for this reason “we need more breadth, more balance, and more doubt”. ( Heath, 2016) You may believe, like us, in the importance of the Humanities in medical education or you may not….but please let your voice be heard.

One hundred years ago, Osler stated, “{Science and Humanities are} twin berries on one stem, grievous damage has been done to both in regarding {them}….in any other light than complemental”. ( Osler, 1919)

We look forward to reading your thoughts on this subject and we accept all forms of publications: research and descriptive papers, opinion pieces and personal views.

## Notes On Contributors

**Mr. Jonathan McFarland** is the Head of Academic Writing at Sechenov First State Medical University in Moscow, and a member of their international faculty. He currently holds the position of President of The Doctor as a Humanist Association, a new Association, which held its first international symposium in October 2017, and which aims to promote and develop internationally the concept of the humanities within undergraduate and postgraduate healthcare education.

**Professor Irina Markovina** is Director of Institute of Linguistics and Intercultural Communication at Sechenov University (Sechenov First Moscow State Medical University). Her interests lie in Psycholinguistics, and she is a representative of the Russian School of Psycholinguistics as well as an Editorial Board member of the peer-reviewed Russian journal “Problems of Psycholinguistics”. She is also committed to developing the English language environment at Sechenov and in other medical universities.

**Professor Trevor Gibbs** is an Independent Consultant in Medical Education and Primary Care development. He is also AMEE International Development Officer and Associate Editor of MedEdPublish. His interests lie in curriculum transformation and development, the Social Accountability of medical schools and teaching, learning and assessment methodologies.

## Declarations

Mr. Jonathan McFarland, Professor Irina Markovina and Professor Trevor Gibbs are all guest Theme Editors for the AMEE MedEdPublish themed issue for Humanities in Medical Education.Trevor Gibbs is Associate Editor of MedEdPublish.

## References

[ref1] BeSST (2018) A Core Curriculum for Sociology in Undergraduate Medical Education. Available at: http://www.besst.info/publications( Accessed: 4 July 2018).

[ref2] Frenk J. Chen L. Bhutta Z. Cohen J. (2010) Health professionals for a new century: transforming education to strengthen health systems in an interdependent world. The Lancet. 376(9756), pp.1923–1958. 10.1016/S0140-6736(10)61854-5 21112623

[ref3] Heath I. (2016) How medicine has exploited rationality at the expense of humanity: An essay by Iona Heath. BMJ. (Online),355, p.i5705. 10.1136/bmj.i5705 27802938

[ref4] Hurwitz B. and Dakin P. (2009) Welcome developments in UK medical humanities. Journal of the Royal Society of Medicine. 102(3), pp.84–85. 10.1258/jrsm.2009.080383 19297643 PMC2746844

[ref5] Kleinman A. (1988) The illness narratives: suffering, healing and the human condition. New York: Basic Books, USA. 10.1097/ACM.0000000000001864 28952997

[ref6] Klugman C. M. (2017) Medical Humanities Teaching in North American Allopathic and Osteopathic Medical Schools. Journal of Medical Humanities, [Epub AoP]. 10.1007/s10912-017-9491-z 29110114

[ref7] Liao L. (2017) Opening our eyes to a critical approach to medicine: The humanities in medical education. Medical Teacher. 39(2), pp.220–221. 10.1080/0142159X.2016.1231915 27670379

[ref8] Mangione S. Chakraborti C. Staltari G. Harrison R. (2018) Medical Students’ Exposure to the Humanities Correlates with Positive Personal Qualities and Reduced Burnout: A Multi-Institutional U.S. Survey. Journal of General Internal Medicine. Journal of General Internal Medicine. 33(5), pp.628–634. 10.1007/s11606-017-4275-8 29380213 PMC5910341

[ref9] Ofri D. (2018) Yes, Studying the Humanities Might Make You a Better Doctor, SLATE. Available at: https://slate.com/technology/2018/04/doctors-should-study-the-humanities.html( Accessed: 4 July 2018).

[ref10] Osler W. (1919) The old humanities and the new science: The presidential address delivered before the Classical Association at Oxford, May, 1919. Journal of the British Medical Association. 2(3053), pp.1–7. 10.1136/bmj.2.3053.1 PMC2343167 20769536

[ref11] Riggare S. (2018) E-patients hold key to the future of healthcare. BMJ. 360, p.k846. 10.1136/bmj.k846 29483151

