# Supplementary material for: Opening Editorial - The Importance of the Humanities in Medical Education
Source: MedEdPublish (2016). 2018 Jul 9;7:140. [Version 1] doi: 10.15694/mep.2018.0000140.1 (PMC10701849; doi:10.15694/mep.2018.0000140.1)
Supplement: Peer review history

# Reviewer report: Reviewer response for version 1

**DOI:** 10.21956/mep.19607.r29131

**Published:** 2018-10-28

**Authors:** Alice Fornari

**Affiliations:** 1 Donald and Barbara Zucker School of Medicine at Hofstra/Northwell

This review has been migrated. The reviewer awarded 5 stars out of 5 i am so pleased this organization i value is taking this topic seriously with a devoted theme issue-the editorial is reassuring that internationally this is important and valued-now we can only hope role modeling to medical students and trainees will be supported and transpired in educational efforts. May we continue a world-wide interest and future collaborations.

Reviewer Expertise:

NA

No decision status is available

# Reviewer report: Reviewer response for version 1

**DOI:** 10.21956/mep.19607.r29130

**Published:** 2018-08-17

**Authors:** David Taylor

**Affiliations:** 1 Gulf Medical University, Ajman, UAE

This review has been migrated. The reviewer awarded 5 stars out of 5 The issue of humanities in medicine has always been important but has often been neglected in the training of healthcare professionals. In these days of rapid change and increasing knowledge it is important to take a step back and look at whether we are spending an appropriate amount of time and energy on the topic.This is a concise and clear editorial introducing a series of articles on this theme which develop the ideas published by Behavioural & Social Sciences Teaching in Medicine (BeSST), and link them in to a consideration of the literature. The editors of the theme have done a valuable job in gathering the papers together and administering the themed issue, and especially in writing this lucid and informative introduction. In response to the call for advice and support in this area AMEE Guide 122 (Kathleen Kendall and colleagues) will be published in the near future.

Reviewer Expertise:

NA

No decision status is available

# Reviewer report: Reviewer response for version 1

**DOI:** 10.21956/mep.19607.r29135

**Published:** 2018-07-27

**Authors:** Sateesh Babu Arja

**Affiliations:** 1 Avalon University School of Medicine

This review has been migrated. The reviewer awarded 5 stars out of 5 I thoroughly enjoyed reading this commentary. I would like to thank authors for taking up an important issue in the current medical field where humanely relationships are going down with the arrival of many technological tools and the changing trends of politiical, economic, and social contexts. This puts physician-patient relationship at risk. I have some teaching experience in behavioral sciences and Medical Ethics. I believe that we should encourage and incorporate humanism leaning theory in our educational strategies. Leaners should be able to realize oneself and able to understand ones feelings. I also have a strong sense of socio-cultural theory and humanities are inseparable and also as authors quoted regarding the practice of medicine from the ivory tower of the university to communities. For the trainees to understand the emotions, they need to understand the societal factors and cultural context which can influence the health and illness. I did remember that we used to involve in community services and community-based education in our medical school as part of the Social and Preventive Medicine course. We also involved in community services and service learning in other courses and modules longitudinally. These activities can make the learners to understand the societal factors influenceing the health and illness which in turn can help them to understand the emotions that people are going through during the illness. This could be economic effects or effects on the family or family members during the periods of illness. Thank you for such a wonderful commentary.

Reviewer Expertise:

NA

No decision status is available

# Reviewer report: Reviewer response for version 1

**DOI:** 10.21956/mep.19607.r29132

**Published:** 2018-07-12

**Authors:** muiris houston

**Affiliations:** 1 TCD

This review has been migrated. The reviewer awarded 5 stars out of 5 Thank you for a stimulating editorial for this theme issue. There is most definitely an unmet need to bring the humanities to a more central position within medical education. It may be a soft topic compared to others but it needs to be “hardened” by incorporating it into the core curriculum.There are many fine programmes out there but too often they languish on the periphery in elective or special study modules. (Hats off to Univ Western Australia for reversing this)Perhaps we could use this publishing opportunity to develop a “how to” exercise aimed at mainstreaming medical humanities ?Dr Muiris HoustonAdjunct Prof Narrative MedicineTrinity College Dublin

Reviewer Expertise:

NA

No decision status is available

# Reviewer report: Reviewer response for version 1

**DOI:** 10.21956/mep.19607.r29134

**Published:** 2018-07-12

**Authors:** Yingzi Huang

**Affiliations:** 1 The First Affiliated Hospital, Sun Yat-sen University

This review has been migrated. The reviewer awarded 5 stars out of 5 Thanks for such an inspiring and interesting theme in a timely manner. Technology nowadays overwhelms people in medicine more and more with fashionable words like AI, big data etc, while humanities in medical education is in danger of being ignored. China is no exception as the second biggest economy with rocketing development since decades ago. A disruptive system without effective primary care has worsen the situation, a case in point is the tension between doctors and patients in China, where people start to reflect on our system and hopefully a consensus is reached that one of the fundamental solutions must be humanities. As an insider, I clearly see both sides of the story. What patients want in China might not necessarily equal what they need. But based on the consideration of information asymmetries, doctors can never avoid the responsibility to readjust their patients’ expectation and ‘educate’ them into rational decision and judgement of their own health, since who else can ever get the job done? To tackle the question of ’how exactly’, one of the weapons for both doctors and patients is humanities, which fully embrace the definition of uncertainties in medicine and diversity in human being, which is why we have to develop humanities in medical education before it’s too late. It’s bound to be difficult but worth the commitment in this field, where eventually trust can be built and our future doctors won’t be any more afraid to ‘wrestle with ambiguity and nuance’ for our patients. It’s a very important theme and I look forward to enjoying reading more thought provoking articles.

Reviewer Expertise:

NA

No decision status is available

# Reviewer report: Reviewer response for version 1

**DOI:** 10.21956/mep.19607.r29129

**Published:** 2018-07-10

**Authors:** Sandra Carr

**Affiliations:** 1 The University of Western Australia

This review has been migrated. The reviewer awarded 4 stars out of 5 Thank you for this well written and thoughtful piece. The humanities have be utilised in the education of health professions for as long as we have been educating health professionals! It is important for us all to reflect on that periodically and this special issue will assist in this. As well as increasing understanding of personal values and beliefs, the humanities can be the vehicle to generate valuable learning and insight for both undergraduates and practitioners into our own unconscious bias and how these biases influence our health care provision. I also agree that the humanities can assist us to balance the sometimes competing tensions between the need to be clinically competent and the need to be caring and sensitive and sensible health professionals. It will be valuable to see what submissions are received in relation to these specific topic areas. With the shift to a graduate entry medical program, the University of Western Australia has developed an undergraduate major in Humanities for Health and Medicine in its Biomedical Science that will commence from 2019. It will be the first such undergraduate major in Australia and may well be the beginning of a new appreciation of the value of the humanities in ALL undergraduate health professions education.

Reviewer Expertise:

NA

No decision status is available

# Reviewer report: Reviewer response for version 1

**DOI:** 10.21956/mep.19607.r29133

**Published:** 2018-07-09

**Authors:** P Ravi Shankar

**Affiliations:** 1 American International Medical University

This review has been migrated. The reviewer awarded 5 stars out of 5 I look forward with great interest to this edition of MedEdPublish on the Medical Humanities. I read the opening editorial with great interest. The editors succinctly sum up the importance of the humanities in medical education. Even today, medicine still deals with uncertainty and medical students and doctors should learn about dealing with uncertainty. The science of medicine is rapidly advancing and science is increasingly dominating modern medicine. There is an increasing awareness of the importance of the humanities in medical education throughout the world. Initiatives are ongoing in various developing countries to use art in the education of doctors. Literature, art, music, painting and other arts contributes significantly to the development of the future physician. I have been involved with the medical humanities since 2007 first in Nepal and then in the Caribbean and look forward to this issue. I eagerly look forward to contributions especially by authors and educators from the developing world.

Reviewer Expertise:

NA

No decision status is available
